# *Rhipicephalus microplus *salivary gland molecules induce differential CD86 expression in murine macrophages

**DOI:** 10.1186/1756-3305-3-103

**Published:** 2010-11-05

**Authors:** Danett K Brake, Stephen K Wikel, Jason P Tidwell, Adalberto A Pérez de León

**Affiliations:** 1 USDA-ARS Knipling-Bushland U.S. Livestock Insects Research Laboratory, 2700 Fredericksberg Rd, Kerrville, TX 78028, USA; 2 Department of Pathology, University of Texas Medical Branch, 301 University Blvd, Galveston, TX 77555, USA; 3 USDA-ARS, Cattle Fever Tick Research Laboratory, Moore Air Base, Building 6419, 22675 North Moorefield Road, Edinburg, TX 78541, USA

## Abstract

**Background:**

Tick parasitism is a major impediment for cattle production in many parts of the world. The southern cattle tick, *Rhipicephalus *(*Boophilus*) *microplus*, is an obligate hematophagous parasite of domestic and wild animals that serves as vector of infectious agents lethal to cattle. Tick saliva contains molecules evolved to modulate host innate and adaptive immune responses which facilitates blood feeding and pathogen transmission. Tick feeding promotes CD4 T cell polarization to a Th2 profile usually accompanied by down-regulation of Th1 cytokines through as yet undefined mechanisms. Co-stimulatory molecules on antigen presenting cells are central to development of T cell responses including Th1 and Th2 responses. Tick induced changes to antigen presenting cell signal transduction pathways are largely unknown. Here we document the ability of *R*. *microplus *salivary gland extracts (SGE) to effect differential CD86 expression.

**Results:**

We examined changes in co-stimulatory molecule expression in murine RAW 264.7 cells in response to *R*. *microplus *SGE exposure in the presence of the toll-like receptor 4 (TLR4) ligand, LPS. After 24 hrs, CD86, but not CD80, was preferentially up-regulated on mouse macrophage RAW 264.7 cells when treated with SGE and then LPS, but not SGE alone. CD80 and CD40 expression was increased with LPS, but the addition of SGE did not alter expression. Higher concentrations of SGE were less effective at increasing CD86 RNA expression. The addition of mitogen or extracellular kinase (MEK) inhibitor, PD98059, significantly reduced the ability for SGE to induce CD86 expression, indicating activation of MEK is necessary for SGE induced up-regulation.

**Conclusions:**

Molecules in SGE of *R. microplus *have a concentration-dependent effect on differential up-regulation of CD86 in a macrophage cell line activated by the TLR4 ligand, LPS. This CD86 up-regulation is at least partially dependent on the ERK1/2 pathway and may serve to promote Th2 polarization of the immune response.

## Background

Ticks carry a variety of emerging and established vector-borne pathogens of medical and veterinary importance including arboviruses, ehrlichiae, spotted fever rickettsiae, *B. burgdorferi*, relapsing fever borreliae, and babesiae [1,2]. Tick- transmitted diseases also have a significant global impact on livestock production and economic development [3]. The southern cattle tick, *Rhipicephalus (Boophilus) microplus *is a vector of bovine babesiosis and anaplasmosis, which are important diseases in cattle throughout tropical and subtropical regions [4,5]. It is estimated that the domestic livestock industry realizes annual savings totalling over three billion dollars at today's currency rate since *R. microplus *and the closely related species *R*. *annulatus *were eradicated from the United States [6,7]. Increasing resistance to commercially available acaracides among *R. microplus *in Mexico is a concern for the US Cattle Tick Eradication Program and a growing threat to the livestock industry [8-11]. Anti-tick vaccines are an alternative method for the control of *R. microplus*. Bm86-based vaccines represent the first generation of anti-tick vaccines to be commercialized [12]. Identifying new vaccine targets and anti-tick strategies for cattle would benefit greatly from a further understanding of the molecular basis underlying tick-host interactions.

*Rhipicephalus microplus *is one-host tick species that evolved complex repertoires of saliva molecules to facilitate feeding and increase reproductive fitness [13,14]. Tick saliva modulates host responses including, hemostasis, wound healing, pain and itch responses, inflammation, and immune defenses [15,16]. Ticks modulate chemokines, T cells, interferon γ (IFNγ)-induced macrophage activation and production of pro-inflammatory cytokines such as interleukin 1β (IL-1β) and tumor necrosis factor α (TNFα), reactive oxygen intermediates, and nitric oxide production [17-20]. Various studies documented the ability of numerous tick species to down-regulate Th1 cytokines while simultaneously up-regulating Th2 cytokines [16]. Th2 polarization was shown to occur upon mitogen stimulation of murine lymphocytes or splenocytes derived from mice infested with *Dermacentor andersoni, Ixodes pacificus, Ixodes ricinus and Rhipicephalus sanguineus *[21-24]. Several studies using murine systems involved stimulating mixed populations of splenocytes or lymphocytes with broad non-antigen dependent T cell stimulants to examine cytokine changes and T cell proliferative potential. It has been shown in *I. scapularis *and *D. andersoni *that tick infestation and salivary gland extracts reduce antigen specific responses [25,26]. Similar immunosuppressive effects have been reported in bovine models. *R. microplus *infestation has been shown to reduce bovine T and B cell numbers and responsiveness [27]. Furthermore, *R. microplus *alters gene expression at the site of attachment as well as cellular subsets and cytokines involved in the inflammatory process in susceptible *Bos taurus *cattle as compared to resistant *Bos indicus *breeds [28,29]. Additionally, a sphinomyelinase-like enzyme in *I. scapularis *saliva has been identified as having a role in altering CD4 T cell responses towards a more Th2 polarization by using an *in vivo *antigen-specific TCR transgenic adoptive transfer model [25,26].

Tick saliva may directly suppress dendritic cell (DC) differentiation and function [30]. Dendritic cells pulsed with *I. ricinus *saliva drive naïve CD4 T cells towards Th2 differentiation [31]. In addition, *in vitro *dendritic cell maturation and ability to induce CD4 T cell proliferation has been shown to be suppressed by *I. scapularis *salivary gland prostaglandin E_2 _[32]. These host evasion strategies alter the immune response to a more Th2 polarization which benefits transmission of tick-borne pathogens that would be counteracted by host Th1 mediated defenses [33].

The mechanisms by which tick saliva alters antigen presenting cell (APC) function are poorly understood. APCs express co-stimulatory molecules CD80 and CD86 and up-regulate expression of these molecules upon activation. Their binding with CD28 is required for T-cell activation in addition to TCR engagement with the cognate antigenic peptide-MHC class II complex [34]. Although structurally related, CD80 and CD86 are distinct glycoproteins expressed on professional APCs such as dendritic cells (DCs), B cells and macrophages [35]. CD80 and CD86 are known to modulate Th1/Th2 cytokine profiles [36-38]. While CD80 preferentially favors Th1 type T cell differentiation, CD86 augments IL-4 production and overall Th2 type T cell responses [37,39,40]. CD86 was shown to be differentially regulated by various cytokines including Th2 promoting IL-4, via activation of MAP kinase and Stat6 [41]. Moreover, TLR signals and the MAPK pathway also control cytokine release during the activation and effector phases of adoptive immune responses [42,43]. Therefore SGE may act to manipulate one or several of these pathways to alter APC responses. In this study, effects of *R. microplus *SGE on the regulation of co-stimulatory molecule expression were examined in the murine macrophage cell line RAW 264.7.

## Results

### Changes in Co-stimulatory molecule expression by SGE

**Figure 1:**
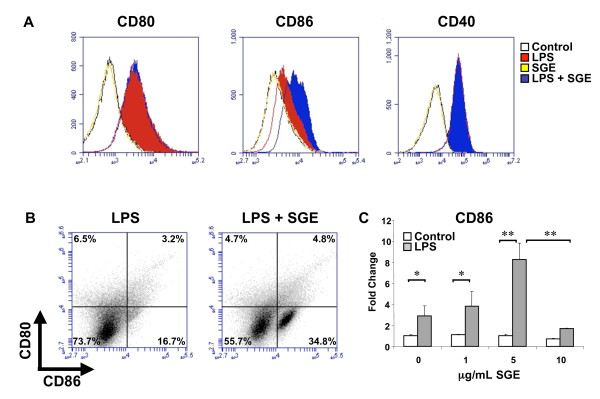
**Cell surface expression of co-stimulatory molecules in response to SGE**. RAW 264.7 cells were stimulated for 1 hr with 5 μg/mL SGE followed by 24 hrs of 100 ng/mL LPS. Cells were then analyzed for co-stimulatory molecule expression by flow cytometry (A & B) or by real-time PCR with varying concentrations of SGE (C). Flow cytometry images are representative of 3 independent experiments. *P < 0.05, ** P < 0.01.

**Figure 2:**
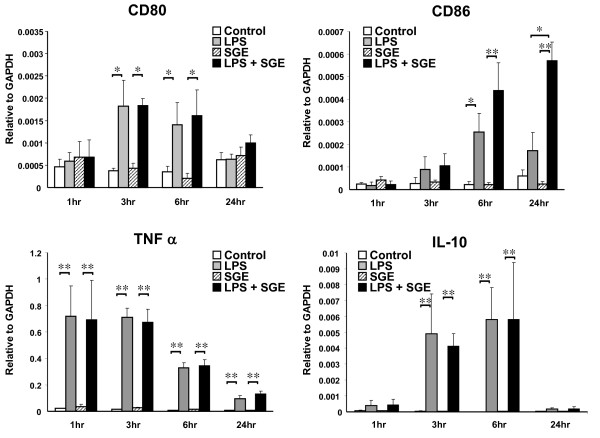
**Relative message expression of co-stimulatory molecules and cytokines over 24 hrs**. RAW 264.7 cells were unstimulated or stimulated for 1 hr with 5 μg SGE followed by 1, 3, 6 or 24 hrs of 100 ng/mL LPS or no LPS. Total RNA was extracted and real-time PCR performed to measure CD80, CD86, IL-10 and TNFα message levels. N = 3-5 independent experiments. * P < 0.05 ** P < 0.01.

Co-stimulatory molecule expression of RAW 264.7 cells was assayed by flow cytometry after 24 hrs of treatment with or without 5 μg/mL of SGE from adult female ticks fed on cattle for 3 days and 100 ng/mL of LPS. Both CD80 and CD40 were up-regulated in the presence of LPS, but addition of SGE did not alter this expression (Figure 1A). LPS with 1 hr pre-treatment of SGE significantly (P ≤ 0.05) increased CD86 expression as compared to LPS alone, SGE alone or untreated cells. Cells co-stained for both CD80 and CD86 showed an increase in the CD86 positive population from 16.7% ± 5.9% SEM with LPS alone to 34.8% ± 5.8% with LPS in the presence of SGE (P < 0.05) (Figure 1B). CD86 RNA expression after 24 hrs of LPS stimulation with 0, 1, 5, or 10 μg/mL of SGE indicate that regulation of CD86 is concentration dependent. 10 μg/mL of SGE show reduced capacity to increase CD86 expression as compared with 5 μg/mL (P < 0.01) (Figure 1C). Co-stimulatory molecule and cytokine mRNA expression was measured at 1, 3, 6 and 24 hrs after LPS stimulation (Figure 2). No significant differences in CD80, TNF-alpha or IL-10 transcripts were detected between LPS alone and LPS in combination with 5 μg/mL SGE. However, CD86 mRNA expression was significantly increased in LPS with SGE group at 24 hrs when compared to LPS alone, SGE alone, or unstimulated cells. Taken together, these data indicate SGE synergizes with LPS to specifically up-regulate CD86 cell surface expression.

### Inhibition of MEK prevents up-regulation of CD86

**Figure 3:**
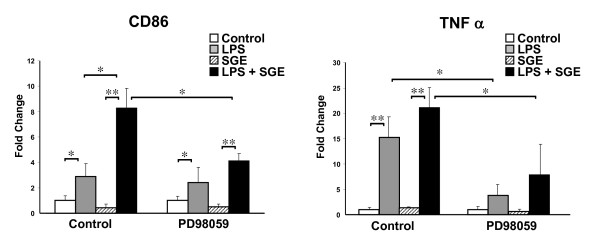
**Inhibition of SGE mediated upregulation of CD86 by MEK inhibitor, PD98059**. RAW 264.7 cells were left untreated or treated with 50 μM PD98059 for 1 hr. Cells were then stimulated with or without 5 μg/mL SGE for 1 hr followed by 24 hrs of 100 ng/mL LPS or no LPS. RNA was extracted and real-time PCR performed to assay CD86 and TNFα message levels. N = 3 independent experiments. * P < 0.05 ** P < 0.01.

The ERK1/2 signaling pathways were blocked pharmacologically by addition of the MEK inhibitor, PD98059. RAW 264.7 cells were treated with 50 μM PD98059 for 1 hr prior to addition of 5 μg/mL SGE for 1 hr followed by 24 hrs of SGE and LPS stimulation. Changes in CD86 and TNFα message were measured by real-time PCR at the 24 hour post-stimulation time point. Addition of PD98059 significantly inhibited CD86 up-regulation by LPS with SGE, but not LPS alone (Figure 3). PD98059, a known inhibitor of LPS-induced TNFα gene expression, did significantly inhibit increases in TNFα message of both LPS and LPS with SGE. This indicates increases in CD86 expression by SGE may be partially dependent on the ERK1/2 pathways.

## Discussion

Modulation of host immune responses by ticks is important for successful blood feeding and facilitation of transmission of tick-borne pathogens in susceptible hosts [16]. This study is the first to examine changes in co-stimulatory molecule expression of antigen presenting cells induced by the SGE of adult female *R. microplus*. We show that at low physiologic concentrations of SGE, CD86 is up-regulated in a murine macrophage cell line. Previously, it was demonstrated that the number of *R. microplus *ticks infesting a host can modulate the antibody response to tick saliva; specifically low to moderate levels of infestation promoted an IgE response where as high infestation showed increases in IgG responses [44]. In the presence of IL-4, a Th2 cytokine, CD86 has been shown to promote IgE synthesis in human B cells [45]. *R. microplus *tick infestation numbers and host breed susceptibility can also alter accumulation of basophils, eosinophils and expression of vascular adhesion molecules involved in immune cell recruitment to sites of infestation [46]. Basophils can promote Th2 responses by IL-4 production and both basophils and eosinophils express CD86 and could be targets of salivary gland molecules [47,48]. Our data show a SGE concentration-dependent effect on CD86 up-regulation, which may indicate the potential of bi-modal responses to differing levels of tick salivary proteins in the skin microenvironment and systemic responses. Previous reports show that saliva from adult *R. sanguineus *females fed for seven days and containing high protein concentrations of 64 μg/mL can inhibit differentiation and maturation of murine bone-marrow-derived dendritic cells including CD80 and CD86 expression [30]. It is well established in blood feeding arthropods that salivary gland gene expression profiles change during feeding and these changes in salivary gland gene expression may have differential effects on immune cell responses [16,49-51].

Ticks tend to modulate host immune responses away from a Th1 profile (measured by decreased IFNγ) and towards a Th2 phenotype (measured by increased IL-4) [22,25,30]. In addition, this Th2 response appears to facilitate pathogen transmission [52,53]. The ability of *I. ricinus *saliva pulsed dendritic cells to drive a Th2 response was initially reported using 15 μg/mL saliva from females fed for 5.5 days [23,31]. In the presence of IL-1β, these DC's showed increased CD80 and CD86 expression and stimulated IL-4 production and priming of naïve CD4 T cells towards Th2 differentiation. Prostaglandin E_2 _from *I. scapularis *saliva showed a trend to increase CD86 expression of bone-marrow derived DC stimulated with LPS, while it inhibited IL-12 and TNFα protein expression in culture supernatants [32].We did not observe changes in IL-10 or TNFα mRNA expression at *R. microplus *SGE concentrations tested in RAW 264.7 cells. *Rhipicephalus microplus *is a metastriate tick and a member of the sub-family most phylogenetically distant from the prostriate *Ixodes *ticks [16]. Salivary gland transcriptomes of prostriate and metastriate species have different proteins repertoires that might be classified into similar functional families [13,54] representing convergent evolution of blood feeding strategies.

Identification and characterization of components in tick saliva responsible for Th2 responses would present targets for vaccine strategies to reduce tick burden and offer alternative eradication strategies. The first tick molecule identified to drive IL-4 response by host CD4 T cells was a spingomyelinase-like enzyme in *I. scapularis *[55]. The authors speculate that the structure could potentially bind Toll-like receptors (TLRs) or other pathogen associated molecular pattern (PAMP) binding molecules. Activation of TLRs is necessary for optimal activation of APCs to initiate and polarize adaptive immune responses against invading pathogens [56,57]. TLR4 deficient mice have reduced CD86 expression on DCs and a reduced ability to promote Th2 cytokines and allergen-specific IgE levels [58]. In our study we used ultrapure LPS shown to signal specifically through TLR4. SGE alone did not affect macrophage cytokine or co-stimulatory molecule expression, but synergized with LPS to increase CD86. In our study we used an immortalized *in vitro *homogenous cell population, devoid of the potential for cross-signalling between heterogeneous populations of immune cells. Further studies are required to examine how SGE affects a complex heterogeneous population of primary murine or bovine immune cells. The authors hypothesize that in a complex skin microenvironment saliva proteins act on a heterogeneous population of cells in concert with other danger signals, endogenous or exogenous, to signal in part, through TLRs to program APCs towards Th2 responses.

IL-4 is primarily involved in promoting the differentiation and proliferation of T helper 2 cells[59]. IL-4 can also act on APCs to polarize them during an active infection and it has been shown to up-regulate CD86 on human alveolar macrophages via ERK1/2 and JAK/STAT6 pathways [41]. Very few studies have examined signal transduction pathways affected by tick saliva molecules. ERK1/2 pathways control cell survival and differentiation [60]. Addition of the MEK inhibitor, PD98059, prior to SGE and LPS treatment, inhibited SGE induced CD86 up-regulation in this study. This indicates that up-regulation of CD86 by SGE may be partially dependent on ERK1/2 pathways. Alteration of CD86 expression may function to promote salivary gland molecule driven Th2 responses, potentially increasing pathogen transmission.

Further studies could examine whether the bioactive factor(s) in SGE act synergistically with IL-4 via ERK1/2 pathways to regulate CD86 expression and subsequent Th2 polarization. This is the first study examining signal transduction pathways affected by *R. microplus *SGE. Previously LPS-induced p38 and ERK phosphorylation was reduced in bone-marrow derived DCs treated with ~40 μg of saliva protein/mL of fully engorged *R. sanguineus *ticks [61]. In conjunction with our findings, this further supports the hypothesis that tick salivary gland molecules may have concentration-dependent effects on local and systemic immune responses.

## Conclusions

Molecules in SGE of *R. microplus *have a concentration-dependent effect on differential up-regulation of CD86 in a macrophage cell line activated by the TLR4-ligand, LPS. This CD86 up-regulation is at least partially dependent on the ERK1/2 pathway and may serve to promote Th2 polarization of the immune response.

## Methods

### Isolation of Tick Salivary Glands

The Deutch strain of *R*. *microplus *used as the source of ticks for this study originated from samples collected in Webb County, TX during an outbreak in 2001. The Deutch strain has been maintained by standard rearing practices at the USDA-ARS Cattle Fever Tick Research Laboratory at Moore Field, TX. The ticks and calves were determined free of *Babesia bovis *and *Babesia bigemina *as described previously [62]. Unfed larvae were placed in patches, one on each side of a stanchioned calf, and allowed to feed following protocols approved by the Institutional Animal Care and Use Committee of the USDA-ARS Knipling-Bushland Livestock Insects Research Laboratory. Upon final ecdysis, adults were allowed to feed for three days and then separated by sex, surface cleaned with 70% ethanol and dissected. Salivary glands were removed and placed into sterile-filtered 0.15 M, Dulbecco's phosphate buffered saline (PBS) (Sigma, St. Louis, MO), pH 7.2 held on ice. Salivary glands were sonicated at 55 kHz for 1 minute on ice and centrifuged at 14,000 × g for 20 minutes at 4°C. The supernatant was collected as salivary gland extract. Protein concentration was determined by Pierce BCA (bicinchoninic acid) Protein Assay (Thermo Scientific, Rockford, IL). The SGE was separated into 20 μl aliquots and subsequently frozen at -70°C and thawed no more than twice.

### Cell Culture

Murine RAW 264.7, monocyte/macrophage-like cells, Dulbecco's Modified Eagle's Medium (DMEM) supplemented with 4 mM L-glutamine, 4,500 mg/L glucose, 1 mM sodium pyruvate,1500 mg/L sodium bicarbonate, and 100 U/mL streptomycin/penicillin were obtained from the American Type Culture Collection (ATCC, Manassas, VA). Cells were cultured in DMEM supplemented with 10% FBS (Atlanta Biologicals, Norcross, GA) and passaged 1:4 by cell scraping with fresh media every 2 days. Cells were subcultured into 12 or 24 well plates overnight to a confluency of 60-80%. Cells were treated with 5 μg/mL of SGE for 1 hr prior to addition of 100 ng/mL of Ultrapure; E coli 0111:B4 lipopolysaccride (LPS), (Fisher Scientific, Pittsburg, PA) for 1, 3, 6 or 24 hrs prior to RNA extraction or flow cytometric analysis. For inhibition studies, 50 μM of MEK inhibitor PD98059 were added for 1 hr prior to addition of SGE.

### Flow Cytometry

RAW 264.7 cells were treated for 1 hr with 5 μg/mL of SGE followed by 24 hrs of 100 ng/mL LPS. Cells were then washed 2× with PBS and immunolabeled with 1 μg of the following antibodies for 30 min at 4°C: CD40 clone: 1C10, CD86 clone: GL1, and CD80 clone: 16-10A1, or non-specific rat or Armenian hamster IgG isotypes to assess background fluorescence (eBioscience, San Diego, CA). Cells were washed 3× with PBS, resuspended in 400 μl PBS, and analyzed on an Accuri C6 Flow Cytometer. Data was analyzed by CFlow Plus software (Accuri Cytometers, Ann Arbor, MI).

### Real-time PCR

RAW 264.7 cells were treated for 1 hr with 5 μg/mL of SGE followed by 100 ng/mL LPS. After 1, 3, 6 or 24 hrs of LPS treatment total RNA was extracted by spin column centrifugation using RNAeasy Mini Kit (Qiagen, Valencia, CA). RNA concentration was determined using a NanoDrop spectrophotometer (Thermo Scientific, Wilmington, DE) and RNA quality was analyzed by agarose gel electrophoresis. Synthesis of cDNA was performed with Superscript III First-Strand Synthesis System for RT-PCR (Invitrogen, Carlsbad, CA), using 500 ng of total RNA and random hexamer primers. For the amplification of specific mRNA, inventoried 20× TaqMan MGB probe-primer sets for CD80, CD86, TNF, IL-10 and GAPDH, was purchased and added to cDNA and 2× TaqMan Universal PCR Master Mix (Applied Biosystems, Foster City, CA). PCR was performed in a CFX96 Real-Time PCR Detection System (BioRad, Hercules, CA) using the following thermal settings: one cycle of 2 min at 50°C followed by 8 min at 95°C, and 40 cycles of 15 s at 95°C, 60 s at 60°C. All reactions were performed in duplicate. Relative mRNA expression was calculated by comparative *C*_t_-method. GAPDH was used as the endogenous control [63].

### Statistics

Results are expressed as means ± SE. Significant differences between means were determined using unpaired Student's t-tests or two-way analysis of variance (ANOVA) with P < 0.05 considered statistically significant.

## Competing interests

The authors declare that they have no competing interests.

## Authors' contributions

DKB, SKW, and AAPL conceived and designed the experiments; DKB and JPT performed the experiments and analysed the data. Manuscript was primarily written by DKB with assistance from SKW, JPT and AAPL. All authors read and approved the final manuscript.
